# A comparison of paediatric hypertension clinical practice guidelines and their ability to predict adult hypertension in an African birth cohort

**DOI:** 10.1038/s41371-022-00709-6

**Published:** 2022-06-14

**Authors:** A. Craig, L. J. Ware, W. Mapanga, S. A. Norris

**Affiliations:** 1 SAMRC/Wits Developmental Pathways for Health Research Unit, Faculty of Health Sciences, University of the Witwatersrand, Johannesburg, South Africa; 2 DSI-NRF Centre of Excellence in Human Development, University of the Witwatersrand, Johannesburg, South Africa; 3 Global Health Research Institute, School of Health and Human Development, University of Southampton, Southampton, UK

**Keywords:** Hypertension, Risk factors

## Abstract

It remains unclear which paediatric hypertension clinical practice guideline (CPG) should be applied in an African population. We, therefore, aimed to compare commonly used CPG (2017 AAP, 2016 ESH, 2004 Fourth Report) developed in high-income countries for use in South African children at four paediatric ages (children: 5 years, 8 years; adolescents: 13 years, 17 years) to determine which best predicts elevated blood pressure (BP) in adulthood (22 years, 28 years). Moreover, the sensitivity, specificity, positive predictive value (PPV) and negative predictive value (NPV) for each specific paediatric CPG was calculated across the age points. The 2017 AAP definition identified more children and adolescents with hypertension when compared to the 2004 Fourth Report and 2016 ESH guidelines. In computed hazards ratios, ages 8 years to 17 years, all three paediatric CPG significantly predicted the risk of elevated BP in young adulthood (*p* ≤ 0.032). However, sensitivity to predict elevated BP at age 22 years for all CPG was generally low (17.0%–33.0%) with higher specificity (87.4%–93.1%). Sensitivity increased at age 28 years (51.4%–70.1%), while specificity decreased (52.8%–65.1%). Both PPV and NPV at both adult age points varied widely (17.9%–79.9% and 29.3%–92.5% respectively). The performance of these paediatric CPG in terms of AUC were not optimal at both adult age points, however, the 2017 AAP definition at age 17 years met an acceptable level of performance (AUC = 0.71). Our results, therefore, highlight the need for more research to examine if an African-specific CPG would better identify high-risk children to minimise their trajectory towards adult hypertension.

## Introduction

Hypertension is a renowned modifiable risk factor for the development of cardiovascular disease (CVD) [1], which is established early in life [2] and affects over 1 billion individuals globally [3]. Until recently, hypertension affected mainly affluent regions of the world, however, low and middle-income countries (LMIC) now account for two-thirds of the global prevalence, which may be attributed to scarce prevention and treatment strategies [4].

Epidemiologically designed longitudinal studies have shown childhood blood pressure (BP) tracks into adulthood [5–7]. It is, therefore, necessary to identify those young individuals who are at high-risk to implement the necessary intervention strategies for the early prevention of CVD. It is believed that these strategies are sufficient models for tackling CVD through prevention and health promotion, aimed at altering an individual’s lifestyle and behavioural habits [8]. Several meta-analyses for the prevention of hypertension found that interventions to reduce salt intake [9] and encourage weight loss through physical activity [10] and a low-calorie diet [11] showed small statistical reductions in BP over time. Presumably the goal of the World Health Organisation’s (WHO) “Global Action Plan for the Prevention and Control of Non-communicable Diseases (NCDs) 2013-2020” will demonstrate the benefits of reducing alcohol ingestion and smoking on premature mortality by the year 2025 [12]. In recognising this, the Fourth Report on the Diagnosis, Evaluation, and Treatment of High Blood Pressure in Children and Adolescents (NHBPEP) (2004 Fourth Report) [13], the American Academy of Pediatrics (2017 AAP) [14] and the European Society of Hypertension (2016 ESH) [15] among others, have provided age, sex, and height specific BP guidelines to allow for the identification of high-risk children.

With the increasing interest surrounding the effect of childhood hypertension and cardiovascular risk in later life, evidence has revealed that hypertension in children is not as uncommon as previously believed, and in most cases represents cardiovascular risk in young adulthood [16]. Adolescents with previous high childhood BP are also more likely to present with persistent hypertension in adulthood [17]. Furthermore, hypertensive youth are also susceptible to accelerated vascular ageing [18] resulting in premature structural and functional alteration within the arterial wall enhancing early onset CVD [19]. Thus, the identification and treatment of early onset hypertension are paramount.

Several population-based studies in several countries (including China, India, Iran, Korea, Poland, Tunisia, and the United States (US)), have prospectively examined the usefulness of these high-income countries’ paediatric clinical practice guidelines (CPG) in identifying high-risk individuals [20, 21]. Certainly, adult hypertension CPG are frequently applied in LMIC settings. For example, systolic BP (SBP) above 140 mmHg (as commonly applied in North America and the European region) has been shown to associate with 6-year mortality in adults from LMIC [22], thus illustrating that a high-income country BP guideline can perform in LMIC adult populations. Whether this can also be replicated in children and more specifically in African children, is unclear and warrants further exploration. We, therefore, aimed to compare three commonly used childhood CPG at four paediatric ages (children 5 years and 8 years; adolescents 13 years and 17 years) to determine which best predicts elevated BP in adulthood (22 years and 28 years) in a South African cohort.

## Methodology

The longitudinal Birth-to-Twenty Plus (Bt20) Cohort enrolled 3273 babies born between April and June 1990 in Soweto-Johannesburg (South Africa) and who remained residents in the area for 6 months. Socioeconomic, personal, and familial information about physical and psychological health and well-being have now been recorded 23 times since birth. The study population and protocol are described elsewhere [23]. Briefly, the study was designed to assess young childhood and adolescent growth, development, and overall health, following the demographic transition in South Africa.

All data presented was collected from participants in the longitudinal birth cohort study Bt20. At the time of recruitment at birth in 1990, the cohort demographically represented babies of long-standing family residents of urban Soweto, however, over time became less representative as migrant families moved into Soweto-Johannesburg from other parts of South Africa and Africa. Blood pressure was measured in this cohort at different data collection waves across the 30 years. For this study we have selected all available BP data from childhood (5 years; *n* = 1235 and 8 years; *n* = 1321), adolescence (13 years; *n* = 1619 and 17 years; *n* = 1853) and adulthood (22 years; *n* = 1541 and 28 years; *n* = 917) – mixed cross-sectional longitudinal design. While overall absolute attrition due to loss to follow-up (death, no longer traceable, requested to be removed from the study) has been low in this cohort (approximately 35.0%), not all active participants were seen in each data collection wave for a variety of reasons. The most common cause was although we were able to establish contact with the participant and caregiver, they did not present themselves during the specific time-period of data collection despite multiple attempts. Consequently, some participants are not seen in one wave but are seen in another. Sensitivity analyses indicated that the only socio-demographic indicators that differed between participants who were screened versus those who were not screened at that time point was a lower household socio-economic status and maternal education. As the focus of this paper is to assess the ability of different CPG to predict adverse BP in adulthood, in statistical analyses all participants where BP measures were obtained within the targeted child and adolescent age point (5 years; 8 years; 13 years and 17 years) and in adulthood (22 years and 28 years) were included (5 years and adulthood; *n* = 832; 8 years and adulthood; *n* = 879; 13 years and adulthood; *n* = 1125 and 17 years and adulthood; *n* = 1422).

The study was conducted in line with the ethical principles of the Declaration of Helsinki [24] and obtained approval from the Human Research Ethics Committee (Medical) of the University of the Witwatersrand (South Africa) (Ref: M190263). All participants were fully informed about the objectives of the study and written informed consent/assent was obtained from each adult and child participant respectively.

### Measurements

Height in cm was measured with a stadiometer (Holtain, United Kingdom) according to specific guidelines and collected using standard protocols [25]. Prior to the measurement of BP, participants were required to remain in a seated position with uncrossed legs for 5-minutes. Three BP measurements were obtained at age 5 years using the automated Dinamap Signs monitor 1846SX (Critikon, USA) and at age 8 years and above using an OMRON M6 oscillometric BP monitor (OMRON HealthCare Co., LTD. Kyoto, Japan). Both devices have been validated for use in children [26]. Using an appropriately sized BP cuff, brachial BP was measured on the left upper arm at 2-minute intervals. The first BP measure was discarded while the mean of the second and third measures were used in statistical analyses. Systolic BP (SBP) and diastolic BP (DBP) were captured from each measurement. Mean arterial pressure (MAP) was subsequently calculated using the formula: [(2 x DBP) + SBP] / 3 [27].

Blood pressure status of the children and adolescent participants (normotensive, pre-hypertension, and hypertension) was classified using specific age, sex, and body height reference standards according to the three paediatric CPG (2004 Fourth report [13], 2017 AAP [14] and 2016 ESH [15]; Table 1). Adult BP status was classified using the South African hypertension practice guideline 2014 (normal: <120 mmHg/<80 mmHg; pre-hypertension:130–139 mmHg/80–85 mmHg and hypertension: >140 mmHg/>90 mmHg) [28]. For adverse BP as an outcome in adulthood, pre-hypertension and hypertension were grouped as elevated BP.

**Table 1:** Childhood, and adolescent clinical practice guidelines (5–17 years).

Classification guideline	Normal	Pre-hypertension	Hypertension Stage 1	Hypertension Stage 2
The Fourth Report on the Diagnosis, Evaluation, and Treatment of High Blood Pressure in Children and Adolescents [13].	1–13 years: Average brachial SBP and/or DBP < 90^th^ percentile for age, sex and height.	1–13 years: Average brachial SBP and/or DBP ≥ 90^th^ percentile but < 95^th^ percentile.	1–17 years: Average brachial SBP and/or DBP ≥ 95^th^ percentile to < 95^th^ percentile + 12 mmHg for age, sex and height (on 3 occasions).	1–17 years: Average brachial SBP and/or DBP ≥ 95^th^ percentile + 12 mmHg for age, sex and height (on 3 occasions).
American Academy of Pediatrics “Clinical Practice Guideline for Screening and Management of High Blood pressure in Children and Adolescents.” [14].	<13 years: Average brachial SBP and/or DBP < 50^th^ percentile for age, sex and height. Separate chart for gender. Actual height in cm or inches is provided on the chart. ≥13 years: <120/<80 mmHg	<13 years: Average brachial SBP and/or DBP < 90^th^ percentile for age, sex and height. Separate chart for gender. Actual height in cm or inches is provided on the chart. ≥13 years: 120/<80 mmHg to 129–80 mmHg.	<13 years: Average brachial SBP and/or DBP ≥ 95^th^ percentile for age, sex and height. Separate chart for gender. Actual height in cm or inches is provided on the chart. ≥13 years: 130/80 mmHg–139/89 mmHg.	<13 years: Average brachial SBP and/or DBP ≥ 95^th^ percentile + 12 mmHg for age, sex and height. Separate chart for gender. Actual height in cm or inches is provided on the chart. ≥13 years: ≥ 140/90 mmHg
2016 European Society of Hypertension guidelines for the management of high blood pressure in children and adolescents [15].	<16 years: Brachial SBP and/or DBP < 90^th^ percentile. ≥16 years: Brachial SBP and/or DBP 130/85 mmHg.	<16 years: Brachial SBP and/or DBP ≥ 90^th^–95^th^ percentile. ≥16 years: Brachial BP 130/85 mmHg to 139/89 mmHg.	0–15 years: Brachial SBP and/or DBP 95^th^–99^th^ percentile + 5 mmHg. ≥ 16 years: Brachial BP 140/90 mmHg to 159/99 mmHg.	<16 years: Brachial SBP and/or DBP > 99^th^ percentile + 5 mmHg. ≥16 years: Brachial BP 160/100 mmHg–179/109 mmHg.

## Statistical analyses

All statistical analyses were performed using IBM^®^ SPSS^®^ version 27 (IBM Corporation, Armonk, New York). Categorical variables were presented as frequency and percentage. Proportions were determined with cross-tabs with significant differences indicated by Chi-square tests. Hazard ratios were computed at each age interval to predict which paediatric CPG best predicts elevated BP in adulthood. Furthermore, the sensitivity, specificity, positive predictive value (PPV) and negative predictive value (NPV) for each specific paediatric CPG was calculated at specific age intervals. Additionally, as performance measures were assessed across various age points, the area under the receiver operating characteristic curve (AUC) [ranging from 0.5 (fail) to 1.0 (excellent)] was measured to reflect the accuracy of the specific paediatric CPG to predict those adults with and without elevated BP as an outcome.

## Results

Overall, each age interval presented with an equal sex distribution (males: 5 years: *n* = 611 (49.5%); 8 years: *n* = 651 (49.3%); 13 years: *n* = 772 (47.7%); 17 years: *n* = 888 (47.9%); 22 years: *n* = 755 (49.0%) and 28 years: *n* = 442 (48.2%)). The prevalence of normotension, pre-hypertension and hypertension at various age intervals according to each guideline was derived (Tables 2a, b). At age 5 years the 2017 AAP classification gave a > 1.07-fold higher number of hypertensive children than the 2004 Fourth Report and 2016 ESH guidelines. At age 8 years the 2017 AAP classification still gave a > 1.23-fold higher number of hypertensive children than the two other CPG. Likewise in adolescence, this difference was greater with a > 3.33-fold difference using the 2017 AAP guideline compared to the 2004 Fourth Report. In adulthood, pre-hypertension and hypertension prevalences were 9.7% and 5.4% at age 22 years and 36.5% and 12.6% at age 28 years respectively.

**Table 2:** a. Blood pressure status of children and adolescence stratified according to mean age intervals based on specific paediatric clinical practice guidelines (*n* (%)). b Blood pressure status in adulthood of the study population based on the South African hypertension practice guideline 2014 (*n* (%)).

	Normal	Pre-hypertension	Hypertension
**a.**			
**Guideline 1 – Fourth Report (2004)**			
Age 5 years (*n* = 1235)	517 (41.9%)	361 (29.2%)	357 (28.9%)
Age 8 years (*n* = 1321)	598 (45.3%)	473 (35.8%)	250 (18.9%)
Age 13 years (*n* = 1619)	1304 (80.5%)	265 (16.4%)	49 (3.1%)
Age 17 years (*n* = 1853)	1279 (69.0%)	475 (24.7%)	99 (5.3%)
**Guideline 2 – AAP (2017)**			
Age 5 years (*n* = 1235)	359 (29.0%)	282 (22.8%)	594 (48.0%)
Age 8 years (*n* = 1321)	425 (32.2%)	289 (21.8%)	607 (46.0%)
Age 13 years (*n* = 1619)	1409 (87.0%)	50 (3.0%)	160 (10.0%)
Age 17 years (*n* = 1853)	1070 (57.7%)	421 (22.7%)	362 (19.5%)
**Guideline 3 – ESH (2016)**			
Age 5 years (*n* = 1235)	530 (42.9%)	153 (12.4%)	552 (44.7%)
Age 8 years (*n* = 1321)	560 (42.4%)	267 (20.2%)	494 (37.4%)
Age 13 years (*n* = 1619)	1293 (79.9%)	153 (9.5%)	172 (10.6%)
Age 17 years (*n* = 1853)	1558 (84.1%)	213 (11.5%)	82 (4.4%)
**b.**			
Age 22 years (*n* = 1540)	1308 (84.9%)	150 (9.7%)	83 (5.4%)
Age 28 years (*n* = 917)	467 (50.9%)	335 (36.5%)	115 (12.6%)

*n* Number of participants, *Fourth Report* The Fourth Report on the Diagnosis, Evaluation, and Treatment of High Blood Pressure in Children and Adolescents, *AAP* American Academy of Pediatrics, *ESH* European Society of Hypertension.

In determining which paediatric CPG at each age best predicted elevated BP in adulthood (22 years and 28 years) (Table 3), we found that at all paediatric ages, except for the 2004 Fourth Report at 5 years (*p* = 0.19), all three of the paediatric CPG significantly predicted elevated BP in adulthood at age 22 years (*p* ≤ 0.049). Similarly, all paediatric ages, except for age 5 years (*p* ≥ 0.32) significantly predicted elevated BP at age 28 years (*p* ≤ 0.001). Overall, with elevated BP as an outcome at age 22 years, the hazard ratio increased with age in each specific CPG, showing a larger hazards ratio with more significance in older participants (17 years). At different paediatric ages, different CPG performed better in predicting adult elevated BP. At age 8 years, the 2017 AAP guideline showed the best prediction of adult (28 years) elevated BP with adverse BP at age 8 years (β = 1.39; [95% CI 1.21; 1.60]; *p* < 0.001). At age 13 years, the 2016 ESH guideline showed the best prediction of adult (28 years) elevated BP (β = 1.27; [95% CI 1.17; 1.40]; *p* < 0.001), while the 2004 Fourth Report showed the best prediction of adult (28 years) elevated BP (β = 1.30; [95% CI 1.18; 1.42]; *p* < 0.001) at age 17 years respectively.

**Table 3:** Hazards ratio to predict which paediatric clinical practice guideline at each age interval best predicts elevated blood pressure as an outcome in adulthood.

	Age 22 years			Age 28 years		
	β	95% (CI)	*p*-value	β	95% (CI)	*p*-value
**Guideline 1 – Fourth Report (2004)**						
Age 5 years (*n* = 832)	1.13	(0.94; 1.36)	0.19	0.98	(0.86; 1.11)	0.73
Age 8 years (*n* = 879)	1.33	(1.11; 1.60)	**0.002**	1.35	(1.19; 1.54)	**<0.001**
Age 13 years (*n* = 1125)	1.32	(1.17; 1.50)	**<0.001**	1.27	(1.16; 1.39)	**<0.001**
Age 17 years (*n* = 1422)	1.66	(1.46; 1.88)	**<0.001**	1.30	(1.18; 1.42)	**<0.001**
**Guideline 2 – AAP (2017)**						
Age 5 years (*n* = 832)	1.32	(1.06; 1.64)	**0.012**	1.07	(0.94; 1.21)	0.32
Age 8 years (*n* = 879)	1.27	(1.05; 1.55)	**0.014**	1.39	(1.21; 1.60)	**<0.001**
Age 13 years (*n* = 1125)	1.27	(1.13; 1.43)	**<0.001**	1.23	(1.13; 1.35	**<0.001**
Age 17 years (*n* = 1422)	1.86	(1.60; 2.17)	**<0.001**	1.29	(1.18; 1.42)	**<0.001**
**Guideline 3 – ESH (2016)**						
Age 5 years (*n* = 832)	1.21	(1.00; 1.46)	**0.049**	1.00	(0.88; 1.14)	0.98
Age 8 years (*n* = 879)	1.22	(1.02; 1.47)	**0.032**	1.25	(1.10; 1.42)	**<0.001**
Age 13 years (*n* = 1125)	1.39	(1.23; 1.57)	**<0.001**	1.27	(1.17; 1.40)	**<0.001**
Age 17 years (*n* = 1422)	1.45	(1.31; 1.61)	**<0.001**	1.28	(1.17; 1.38)	**<0.001**

*n* Number of participants, *Fourth Report* The Fourth Report on the Diagnosis, Evaluation, and Treatment of High Blood Pressure in Children and Adolescents, *AAP* American Academy of Pediatrics, *ESH* European Society of Hypertension. Bold values denote statistical significance (*p* < 0.05).

A comparison of the performance of the three specific CPG to identify elevated BP in adulthood (22 years and 28 years) is presented in Tables 4a, b. At age 22 years, across all CPG, the thresholds manifested a low sensitivity (17.0–33.0%), a high specificity (87.4–93.1%), a moderately low to high PPV (23.5–79.9%) and corresponding NPV (29.3–88.9%). At age 28 years, the thresholds to identify elevated BP in adulthood indicated that sensitivity increased to a moderate to high range (51.4.0–70.1%), while specificity decreased to a moderate range (52.8–65.1%). The PPV (17.9–77.7%) and NPV (33.6–92.5%) at age 28 years still remained with a broad range from moderately low to high. Furthermore, at both adult outcomes (22 years and 28 years), the performance of these CPG in terms of AUC suggested that only the 2017 AAP definition at age 17 years (AUC = 0.71) met an acceptable level of performance to identify elevated BP in adulthood (22 years), while all other CPG did not perform adequately in identifying elevated BP in adults (AUC ≤ 0.68).

**Table 4:** a. Sensitivity, specificity, predictive value and accuracy of paediatric clinical practice guideline at each age interval to predict elevated blood pressure as an outcome at age 22 years. b. Sensitivity, specificity, predictive value and accuracy of paediatric clinical practice guideline at each age interval to predict elevated blood pressure as an outcome at age 28 years.

	Guideline	Sensitivity (95% CI)	Specificity (95% CI)	Predictive valve (95% CI)		AUC (95% CI)	Absolute adults detected *n* (%)	Absolute adults missed *n* (%)
				Positive	Negative			
**a.**								
Elevated BP & hypertension outcome (Age 5 years)	Fourth (2004)	17.7 (14.5; 21.4)	87.8 (83.7; 91.0)	68.2 (59.4; 76.0)	41.8 (38.2; 45.6)	0.53 (0.46; 0.60)	109 (13.1)	20 (2.4)
	AAP (2017)	18.0 (15.1; 21.3)	91.2 (86.5; 94.4)	84.5 (76.8; 90.0)	29.3(26.0; 32.8)	0.55 (0.48; 0.62)	88 (10.6)	41 (4.9)
	ESH (2016)	18.3 (15.0; 22.0)	88.5 (84.5; 91.6)	69.8 (61.0; 77.4)	42.7 (39.0; 46.4)	0.55 (0.48; 0.62)	90 (10.8)	39 (4.7)
Elevated BP & hypertension (Age 8 years)	Fourth (2004)	20.4 (16.9; 24.3)	89.8 (86.3; 92.5)	71.2 (62.8; 78.4)	47.7 (44.1; 51.4)	0.59 (0.52; 0.66)	99 (11.3)	40 (4.6)
	AAP (2017)	18.3 (15.4; 21.7)	89.8 (85.4; 93.0)	79.9 (72.0; 86.0)	33.2 (29.9; 36.8)	0.59 (0.52; 0.65)	111 (12.6)	28 (3.2)
	ESH (2016)	19.3 (16.0; 23.0)	89.1 (85.3; 92.0)	71.2 (62.8; 78.0)	44.1 (40.5; 47.7)	0.56 (0.49; 0.63)	100 (11.4)	41 (4.7)
Elevated BP & hypertension (Age 13 years)	Fourth (2004)	26.0 (20.7; 32.1)	87.4 (85.1; 89.4)	33.7 (27.1; 41.0)	82.8 (80.3; 85.0)	0.58 (0.51; 0.66)	63 (5.6)	124 (11.0)
	AAP (2017)	27.7 (21.0; 35.4)	86.6 (84.4; 88.5)	23.5 (17.9; 30.4)	88.9 (86.8; 90.7)	0.55 (0.47; 0.62)	44 (3.9)	143 (12.7)
	ESH (2016)	27.6 (22.2; 33.6)	87.9 (85.7; 89.9)	37.4 (30.6; 44.8)	82.3 (79.8; 84.5)	0.61 (0.54; 0.69)	70 (6.2)	117 (10.4)
Elevated BP & hypertension (Age 17 years)	Fourth (2004)	28.8 (24.6; 33.3)	91.1 (89.1; 92.8)	58.7 (51.7; 65.3)	74.4 (71.9; 78.6)	0.68 (0.61; 0.75)	125 (8.8)	88 (6.2)
	AAP (2017)	26.0 (22.5; 29.7)	93.1 (91.0; 94.7)	73.2 (66.7; 78.9)	63.2 (60.4; 65.9)	0.71 (0.65; 0.78)	156 (11.0)	57 (4.0)
	ESH (2016)	33.0 (27.0; 40.0)	88.4 (86.4; 90.1)	34.7 (28.4; 41.6)	87.6 (85.6; 79.6)	0.59 (0.52; 0.67)	74 (6.5)	139 (9.8)
**b.**								
Elevated BP & hypertension (Age 5 years)	Fourth (2004)	51.4 (45.4; 57.4)	52.8 (45.9; 59.6)	58.9 (52.2; 65.1)	45.2 (38.9; 51.6)	0.52 (0.45; 0.59)	145 (29.2)	101 (20.4)
	AAP (2017)	52.2 (46.8; 57.5)	56.4 (48.0; 64.4)	73.6 (67.5; 78.9)	33.6 (27.8; 39.9)	0.54 (0.47; 0.60)	181 (36.5)	65 (13.1)
	ESH (2016)	53.9 (47.0; 60.6)	52.3 (46.3; 58.3)	59.3 (52.9; 65.5)	46.8 (40.5; 53.1)	0.53 (0.47; 0.60)	146 (29.4)	100 (20.2)
Elevated BP & hypertension (Age 8 years)	Fourth (2004)	59.1 (53.2; 64.7)	63.0 (56.3; 69.2)	57.7 (61.6; 73.3)	54.0 (47.8; 60.0)	0.60 (0.53; 0.66)	176 (33.5)	84 (16.0)
	AAP (2017)	56.3 (51.0; 61.4)	65.1 (57.2; 72.2)	77.7 (72.0; 82.5)	40.8 (34.8; 47.0)	0.60 (0.53; 0.66)	202 (38.5)	58 (11.0)
	ESH (2016)	56.7 (51.0; 62.3)	61.0 (54.0; 67.6)	68.1 (62.0; 73.6)	49.1 (42.9; 55.2)	0.57 (0.50; 0.63)	177 (33.7)	83 (15.8)
Elevated BP & hypertension (Age 13 years)	Fourth (2004)	69.9 (61.6; 77.0)	55.8 (51.8; 60.0)	27.2 (22.8; 32.1)	88.7 (85.0; 91.6)	0.59 (0.52; 0.65)	102 (13.4)	273 (35.7)
	AAP (2017)	69.8 (59.4; 78.5)	53.9 (50.0; 57.7)	17.9 (14.2; 22.2)	92.5 (89.4; 94.9)	0.55 (0.49; 0.62)	67 (8.8)	308 (40.3)
	ESH (2016)	68.4 (60.4; 75.5)	55.8 (51.7; 59.7)	28.3 (23.8; 33.1)	87.4 (83.6; 90.4)	0.59 (0.52; 0.66)	106 (13.9)	269 (35.2)
Elevated BP & hypertension (Age 17 years)	Fourth (2004)	66.7 (60.7; 72.2)	59.2 (55.1; 63.1)	43.0 (38.3; 47.9)	79.3 (75.2; 82.9)	0.62 (0.56; 0.69)	182 (21.1)	241 (27.9)
	AAP (2017)	63.0 (57.8; 67.9)	61.5 (57.0; 65.8)	55.1 (50.2; 59.9)	68.9 (64.3; 73.1)	0.61 (0.55; 0.68)	233 (27.0)	190 (22.0
	ESH (2016)	70.1 (61.9; 77.3)	55.2 (51.5; 58.9)	23.9 (20.0; 28.3)	90.2 (87.0; 92.8)	0.57 (0.50; 0.64)	101 (11.7)	322 (37.3)

*n* Number of participants, *AUC* Area under the curve, *Fourth Report* The Fourth Report on the Diagnosis, Evaluation, and Treatment of High Blood Pressure in Children and Adolescents, *AAP* American Academy of Pediatrics and, *ESH* European Society of Hypertension.

## Discussion

We aimed to compare the three most commonly applied international paediatric CPG for hypertension in an African paediatric cohort and to assess the utility of these to predict elevated BP in adulthood. Examining the prevalence of childhood hypertension, we found that there was a higher hypertension prevalence using the 2017 AAP guideline than using either the 2004 Fourth Report or the 2016 ESH guidelines. Across various childhood and adolescent age points, the 2017 AAP definition indeed showed an expected higher hypertension prevalence, which is consistent with the findings from numerous recent cross-sectional studies conducted worldwide [21, 29, 30]. We also found that the prevalence of pre-hypertension decreased across our specific ages with a concomitant upward trend in the prevalence of hypertension [20, 31]. However, contrary to our findings, one US study reported a lower hypertension prevalence when applying the 2017 AAP guidelines in a large paediatric population when compared to the 2004 Fourth Report, presumably due to differences in age range [32].

The most prominent reason why several studies, including ours, have observed disparities in hypertension prevalence when applying the 2017 AAP definition compared with the two other paediatric definitions, could be due to the notable change seen in the revised BP charts adapted by the 2017 AAP guideline, which excludes overweight and obese individuals. Higher levels of adiposity represent one of the most important risk factors for the development of hypertension in children [33]. This exclusion of overweight and obese children in the development of the CPG yields a lower BP cut off value (2–3 mmHg lower) when compared with the original BP charts included in the 2004 Fourth Report. Consequently, in a population sample that includes overweight and obese children and adolescents, the overall prevalence will increase.

Other likely reasons may be due to linear growth of the study population, as height is a major independent determinant of childhood BP classification [34]. Comparing the 2017 AAP and 2016 ESH guidelines, the 2017 AAP definition shows a much broader BP range in shorter individuals (those in the lower 50% height percentile), while this gap tends to fade in taller individuals (those between 90–95% height percentiles) [35]. Consequently, the application of the 2017 AAP guidelines could in theory reclassify those with a shorter stature downward into a lower BP category. When evaluating the growth of the Bt20 study population included in this study, all age points showed on average lower linear growth than that described by age, sex, and mean height percentiles of the WHO [36]. Furthermore, the revised 2017 AAP definition recommends a single BP threshold to define adolescent hypertension (those aged > 13 years) with no adjustments made for age, sex, or height [14]. This static cut off (130/80 mmHg) corresponds to those for adults adapted from the American Heart Association and the American College of Cardiology. The simple method, however, has not performed well in girls and younger adolescents due to low sensitivity values [37]. This, however, did not come as a surprise as prior to the revisions made to the 2017 AAP definition, BP cut-offs for defining hypertension in adolescent girls (50^th^ percentile: 124/79 mmHg (13 years) to 127/81 mmHg (17 years)) were lower than the new recommended single cut-off value [14]. Similarly, in younger adolescents (13–14 years), the BP cut-offs for hypertension across multiple height percentiles were also lower than the single BP cut off. Therefore, this highlights the need to examine the linear growth effect on childhood BP classification in a LMIC setting and explore optimal single cut off points for adolescents.

Considering childhood BP is known to track into adulthood, we further sought to determine how well paediatric CPG predict adverse BP in adults. When looking at the performance of these results, the performance of all three CPG were not optimal in childhood to predict elevated BP in adulthood. Additionally, at age 22 years, our performance results show using paediatric CPG were highly specific (specificity > 86%) yet not sensitive (sensitivity < 35%) in predicting adverse BP in adulthood, although the closer to adulthood the measurement was, the better the prediction (2017 AAP definition showed an AUC value of 0.71 at age 17 years), which may highlight a potential window of opportunity for screening high-risk adolescents. It has been reported that juvenile BP measured in childhood and adolescence strongly predicts BP in mid-life [38] and given that the Bt20 cohort comprises of young adults (<30 years of age), we may expect less predictability. The sensitivity of our performance did, however, moderately increase (<70%) as older adults were assessed (28 years), but specificity at this age point decreased (<65%). Consequently, across all paediatric definitions <13% of children and adolescents who previously were classified as having childhood pre-hypertension or hypertension remained with adverse BP as adults at age 22 years and <39% at age 28 years respectively. At older adult age points, more of the cohort exhibited pre-hypertension (by + 26.8%) and hypertension (by + 7.2%) showing a 3-fold increase in adverse BP in just 6 years, which could have influenced the ability to detect and/or predict adverse BP from earlier measures.

As childhood hypertension is often unrecognised due to symptoms rarely presenting among children, as in adults, and hypertension-related complications usually occur several years after onset [13, 14], identifying high-risk children and adolescents allows for the opportunity to implement low-cost lifestyle changes, which may halt or even reverse the progression of hypertension and reduce early onset cardiovascular alterations. However, as a result, applying the revised 2017 AAP definition has been frequently adopted worldwide, where more children and adolescents are classified as hypertensive, this simple adoption of childhood BP nomograms from a high-income country such as the US may, however, not be best suited for LMIC with diverse population characteristics. These guidelines may therefore introduce unpredicted bias in evaluating childhood BP resulting in a significant over or under-estimation of normotension, pre-hypertension and/or hypertension in children and adolescents. Therefore, the need to explore country or region-specific CPG has gained much focus over the past decade [39–41].

Our findings must be interpreted within the context of the strengths, limitations, and recommendations of this study. This study was the first to compare three commonly used paediatric CPG to assess the prevalence of childhood hypertension and the risk of elevated BP in adulthood in an African cohort. We also acknowledge that our study was limited in the fact that BP measures were only obtained at one data collection timepoint per age interval and not on three separate occasions as per recommended guidelines thus further study is needed to investigate differences in the prevalence of childhood hypertension identified by the three CPG across three successive visits. Also, out of home measurements may increase BP due to white coat effects, even though Bt20 is a birth cohort and participants are very comfortable and familiar with study staff and setting. Our study included urban African participants and may not be representative of a national sample. Furthermore, strategies for preventing hypertension such as studies exploring prediction models are needed which could enable targeted primordial prevention.

In conclusion, our results showed a concerning picture of rising elevated BP (combined pre-hypertension and hypertension) prevalence from childhood into adulthood, which warrants more prevention efforts to be upscaled with appropriate methods and guidelines to support this. Also, we confirmed that the prevalence of hypertension among children and adolescents was higher when the 2017 AAP guidelines were applied than those of the 2004 Fourth Report or 2016 ESH guidelines. All three specific paediatric CPG were found to significantly predict the risk of elevated BP in adulthood at ages 8 years, 13 years and 17 years respectively. This, however, must be interpreted with caution considering the poor predictability performance of these estimates.

### Summary

#### What is known

- Childhood blood pressure (BP) tracks into adulthood.
- It is vital to identify high-risk children and adolescents in order to implement the necessary intervention and prevention strategies to curb early onset cardiovascular disease.
- Age, sex and height specific paediatric clinical practice guidelines (CPG) have been developed to allow for the identification of high-risk children. These guidelines were developed in high-income countries.
- The 2017 American Academy of Pediatrics (2017 AAP) CPG yields a higher hypertension prevalence when compared to other paediatric CPG due to revised BP charts that exclude overweight and obese individuals.

#### What this study adds

- In an African cohort, we confirmed the expected increase in hypertension prevalence when the 2017 AAP guideline was applied.
- We noted that the growth of our African study population across numerous age points showed an on average lower linear growth than that described by the WHO. As height is a major determinant of childhood BP and pediatric CPGs are height specific, our study highlighted the need to examine the linear growth effect on childhood BP classification.
- The adoption of paediatric CPG nomograms from high-income countries may not be best suited for a low-to-middle income setting, due to diverse population characteristics. Our study suggests the need to explore country or region-specific CPG.
- When we explored which paediatric CPG best predicts elevated BP in young adulthood, all three CPG significantly predicted the risk of elevated BP in young adulthood, however, the performance in terms of AUC was not optimal.

## Data Availability

The datasets generated during and/or analysed during the current study are not publicly available but are available from the corresponding author on reasonable request.
